# Low-Dose Computed Tomography Image Super-Resolution Reconstruction via Random Forests

**DOI:** 10.3390/s19010207

**Published:** 2019-01-08

**Authors:** Peijian Gu, Changhui Jiang, Min Ji, Qiyang Zhang, Yongshuai Ge, Dong Liang, Xin Liu, Yongfeng Yang, Hairong Zheng, Zhanli Hu

**Affiliations:** 1 Lauterbur Research Center for Biomedical Imaging, Shenzhen Institutes of Advanced Technology, Chinese Academy of Sciences, Shenzhen 518055, China; pj.gu@siat.ac.cn (P.G.); ch.jiang@siat.ac.cn (C.J.); qy.zhang@siat.ac.cn (Q.Z.); ys.ge@siat.ac.cn (Y.G.); dong.liang@siat.ac.cn (D.L.); xin.liu@siat.ac.cn (X.L.); yf.yang@siat.ac.cn (Y.Y.); hr.zheng@siat.ac.cn (H.Z.); 2 School of Information Engineering, Wuhan University of Technology, Wuhan 430070, China; 3 Shanghai United Imaging Healthcare, Shanghai 201807, China; min.ji@united-imaging.com

**Keywords:** coupled dictionary learning, low-dose CT, random forests, super-resolution

## Abstract

Aiming at reducing computed tomography (CT) scan radiation while ensuring CT image quality, a new low-dose CT super-resolution reconstruction method based on combining a random forest with coupled dictionary learning is proposed. The random forest classifier finds the optimal solution of the mapping relationship between low-dose CT (LDCT) images and high-dose CT (HDCT) images and then completes CT image reconstruction by coupled dictionary learning. An iterative method is developed to improve robustness, the important coefficients for the tree structure are discussed and the optimal solutions are reported. The proposed method is further compared with a traditional interpolation method. The results show that the proposed algorithm can obtain a higher peak signal-to-noise ratio (PSNR) and structural similarity index measurement (SSIM) and has better ability to reduce noise and artifacts. This method can be applied to many different medical imaging fields in the future and the addition of computer multithreaded computing can reduce time consumption.

## 1. Introduction

Computed tomography (CT) uses precisely collimated X-rays, gamma rays, ultrasonic waves, or other types of beams in concert with highly sensitive detectors to sequentially scan individual sections of the human body. CT has a fast scan time and results in clear images. Thus, CT is used in examinations for a variety of diseases. CT scanners are one of the most commonly installed types of medical imaging diagnostic equipment and are widely used in various clinical fields. Various types of radiation can be used for CT; however, radiation can cause certain damage to the patient’s body, such as to the head, which may lead to headaches or insomnia [1]. Therefore, the ideal radiation dose for medical applications should be minimized [2]. Many methods currently exist for reducing radiation doses, such as reducing the voltage, the current, the clinical scanning time and so on. However, these approaches cause increased noise, granularity and serious artifacts in the resulting CT images, which can result in misdiagnoses [3]. Many methods to reduce these disadvantages of low-dose CT images have emerged in the super-resolution field in recent years [4,5,6].

Super-resolution (SR) reconstruction is a classical image recovery technique usually divided into three categories. The first category is the traditional interpolation method [7,8,9]. Simple interpolation methods such as bicubic interpolation can produce a smoother image that achieves a certain denoising effect and preserves edges in the zoomed image but it is powerless for removing artifacts. When dealing with visually complex real images (such as CT images) the effect of traditional interpolation is limited and can even generate artifacts. The second category is based on models [10,11,12,13]. Model-based techniques perform image reconstruction by projecting features of the image based on the degradation process of the simulated image. When a priori knowledge of the model image is effectively applied, these techniques can guarantee the quality of the reconstructed image [10,13]. However, when no a priori knowledge is available, they tend to result in an ill-posed problem because of an insufficient number of low-resolution images. Conversely, using excessive numbers of images in training can lead to long runtimes and lengthy computation.

The third category of SR reconstruction is based on machine learning [14]. Machine learning algorithms learn a nonlinear mapping of a training database consisting of low-resolution (LR) and high-resolution (HR) image pairs to obtain connections between the LR images and HR images [4,15,16,17,18,19,20,21]. In recent years, the academic community has become increasingly interested in implementing SR based on sparse representation methods because this approach robustly preserves image features and suppresses noise and artifacts [15,18,21]. For example, Dong et al. [22] used adaptive sparse domain selection and adaptive regularization to cluster the training data and create a compact dictionary. This approach obtained a good SR result. Yang et al. [15] proposed a novel coupled dictionary training method for SR based on patchwise sparse recovery. Jiang et al. [18] proposed a single CT image SR reconstruction scheme. However, these methods require sparse coding in both the training and inference phases; therefore, their processing speeds are slow. To solve the above problems, Timofte et al. [23,24] proposed an instance-based neighbourhood regression SR algorithm and Samuel et al. [25] proposed a fast and accurate SR method based on a random forest classification mapping relationship.

Random forest (RF) is suitable for the problem framework of local linear multiple regression [26,27,28]. RF has highly nonlinear learning characteristics, is usually very fast during training and evaluation and can easily adapt to inputs consisting of noisy low-resolution images; thus, RF is widely applied in the computer vision field. Inspired by coupled dictionary learning and RF, a similar method to solve the SR of low-dose CT (LDCT) and obtain reconstructed CT images with similar quality to high-dose CT (HDCT) images is proposed here. In addition, during the SR imaging process, a series of iterations are added to improve the quality of the final reconstructed image. The proposed method is also compared with the traditional interpolation method and important indicators are evaluated.

This paper is organized as follows. Section 2 provides background information concerning the related sparse representation and dictionary learning techniques. In Section 3, a random forest-based solution for SR was proposed. Section 4 presents the experimental results. Finally, Section 5 provides discussions and future works and concludes the paper.

## 2. Background

### 2.1. Sparse Representation

According to the principle of compressed sensing [29,30] and sparse representation [31], an image vector x can be represented as a sparse linear combination of a dictionary D and it is mathematically expressed as follows:

(1) $$x=D\alpha \;\mathrm{for}\;\mathrm{some}\;\alpha \in R^{K}\;\mathrm{with}\;{\left|\left|\alpha \right|\right|}_{0}\ll K$$

where α is the sparse representation coefficient and the content ${\left|\left|\alpha \right|\right|}_{0}\ll K$, where K is the dimension of x, represents an image block. The matrix D is a dictionary with K×n dimensions. An overcomplete dictionary, that is, where the number of atoms n is larger than the dimension of the image block K, is often used for sparse representation; the sparse coefficient α can be obtained by an optimized estimation of the cost function. Generally, the cost function is expressed as follows:

(2) $$F\left(\alpha \right)={\left|\left|x-D\alpha \right|\right|}_{2}^{2}+\lambda {\left|\left|\alpha \right|\right|}_{1}$$

where λ is a constant parameter. The sparse representation is extended to the SR problem via the following function:

(3) $$F\left(\alpha \right)={\left|\left|y-HD\alpha \right|\right|}_{2}^{2}+\lambda {\left|\left|\alpha \right|\right|}_{1}$$

where the vector y is the LR image block and H is the sampling matrix. Using the matrix H, the degradation of the geometric shift, blur, or down-sampling operator can be determined for the LR image y. The cost function is minimized as follows:

(4) $$I=\sum_{i}min\left[{\left|\left|y_{i}-D\alpha_{i}\right|\right|}_{2}^{2}+\lambda {\left|\left|\alpha_{i}\right|\right|}_{1}\right]$$

When solving the optimal vector problem in Equation (4), how the dictionary is established is highly important for mapping the LR and HR images.

### 2.2. Coupled Dictionary Learning

The main method for dictionary-based single-image super-resolution was based on coupled dictionary learning. The most effective method was proposed by Yang et al. [15,16]. N samples sampled from the LR and HR images are denoted $X_{L}\in R^{D_{L}\times N}$ and $X_{H}\in R^{D_{H}\times N}$, respectively. The symbols $X_{L}$ and $X_{H}$ represent the LR and HR data matrices, respectively and each column represents a sample $x_{L}$ and $x_{H}$. The coupled dictionary learning method can be defined as follows:

(5) $$min=\frac{1}{D_{L}}{\left|\left|X_{L}-D_{L}E\right|\right|}_{2}^{2}+\frac{1}{D_{H}}{\left|\left|X_{H}-D_{H}E\right|\right|}_{2}^{2}+\Gamma \left(E\right)$$

where $D_{L}\in R^{D_{L}\times B}$ represents the LR dictionaries and $D_{H}\in R^{D_{H}\times B}$ represents the HR dictionaries. The code sparse matrix connecting these two dictionaries is $E\in R^{B\times N}$. The regularization term Γ(E) is usually a sparse specification constraint of E using the $l_{0}$-norm or $l_{1}$-norm.

In Equation (5), in coupled dictionary learning, the mapping relationship between LR and HR image is critical, as defined below:

(6) $$X_{H}=W\left(X_{L}\right)\cdot X_{L}$$

Equation (6) shows that dictionary training can be performed only when the mapping relation function $W\left(X_{L}\right)$ is known. Using a random forest, the method of learning this mapping is discussed below.

## 3. Proposed Reconstruction Method

### 3.1. Mapping Relation Function Learning

This section discusses learning the mapping relation function $W\left(X_{L}\right)$. First, consider a two-paradigm objective function, as follows:

(7) $$\mathrm{argmin}\sum_{n=1}^{N}{\left|\left|X_{H}^{n}-W\left(X_{L}^{n}\right)\cdot X_{L}^{n}\right|\right|}_{2}^{2}$$

According to different basis functions ψ(x), Equation (7) is converted to

(8) $$\mathrm{argmin}\sum_{n=1,\forall j}^{N}{\left|\left|X_{H}^{n}-\sum_{j=0}^{\gamma }W_{j}\left(X_{L}^{n}\right)\cdot \psi_{j}(X_{L}^{n})\right|\right|}_{2}^{2}$$

The goal of this paper is to find the regression matrix $W_{j}\left(X_{L}^{n}\right)$ for each γ+1 basis function. While one option is to choose a linear basis function, such as $\psi_{j}\left(x\right)=x$, a polynomial function, such as $\psi_{j}\left(x\right)=x^{j}$, can also be chosen. Different parameter settings has different effects. In either case, the target linear and nonlinear parameters can be learned through their dependencies.

This paper used random forests to create data dependence. A random forest is a binary tree and multivariate regression is performed using the dimension of the dictionary $D_{H}$; that is, each tree independently separates the data space, the leaf nodes are determined and then, the nodes are overlapped by using multiple trees and multiple forests so that each leaf node learns a linear model:

(9) $$m_{l}\left(x_{l}\right)=\sum_{j=0}^{\gamma }W_{j}^{l}\cdot \psi_{j}\left(x_{L}\right)$$

However, to find all the matrices $W_{j}^{l}$, the regularized least squares problem must be solved, which can be solved by the formula $W^{lT}={\left(\Psi {\left(X_{L}\right)}^{T}\Psi \left(X_{L}\right)+\eta I\right)}^{-1}\Psi {\left(X_{L}\right)}^{T}\cdot X_{H}$. Therefore, all the data are stacked into the matrix $W^{l},\;\Psi \left(X_{L}\right)$ and $X_{H}$ and the user specifies the regularization parameter η. Because all the binary trees are used for prediction during the inference process, the data dependency matrix $W\left(X_{L}\right)$ can be described as follows:

(10) $${\hat{x}}_{H}=m\left(x_{L}\right)=W\left(x_{L}\right)\cdot x_{L}=\frac{1}{T}\sum_{t=1}^{T}m_{l\left(t\right)}\left(x_{L}\right)$$

where l(t) is the leaf node of tree t generated by sampling point $x_{L}$ and T is the number of trees.

### 3.2. Tree Structure Learning

We obtain the leaf node model using Equation (9) and then train the tree to find the optimal solution of the mapping relation function. N samples $\left\{x_{L}^{n},x_{H}^{n}\right\}\in X\times Y$ are taken, where X and Y represent the LR and HR images, respectively. A single random tree is trained by finding the split function and using recursion to segment the training data into disjoint subsets. The split function is

(11) $$\delta \left(x_{L},\Theta \right)=\{\begin{array}{c} 0\;\;r_{\Theta }\left(x_{L}\right)<0\\ 1\;otherwise \end{array}$$

For all internal tree nodes, the split starts at the root node and continues down the tree in a greedy manner until it reaches the maximum depth $\xi_{max}$, at which point the leaf nodes are created.

To find a good parameter Θ for the split function $\delta \left(x_{L},\Theta \right)$, the general method is to sample the random group by a quality metric to obtain the parameter value $\Theta_{k}$ and choose the best one. The quality of the splitting function $\delta \left(x_{L},\Theta \right)$ is defined as follows:

(12) $$Q\left(\delta ,\Theta ,X_{L},X_{H}\right)=\sum_{c\in \left\{Left,Right\right\}}\left|X^{c}\right|\cdot E\left(X_{L}^{c},X_{H}^{c}\right)$$

where Left and Right are the left and right sub-nodes, respectively and |·| is the cardinal operator. According to the split function in Equation (11), two new domains are defined:

(13) $$\left[X_{L}^{Left},X_{H}^{Left}\right]=\left\{\left[x_{L},x_{H}\right]:\delta \left(x_{L},\Theta \right)=0\right\}$$

(14) $$\left[X_{L}^{Right},X_{H}^{Right}\right]=\left\{\left[x_{L},x_{H}\right]:\delta \left(x_{L},\Theta \right)=1\right\}$$

The function $E\left(X_{L},X_{H}\right)$ is used to measure the purity of the data, causing similar data to fall into the same leaf node to achieve the random forest classification goal.

A new regularization expression is thus defined:

(15) $$E\left(X_{L},X_{H}\right)=\frac{1}{N}\sum_{n=1}^{N}\left({\left|\left|x_{H}^{n}-m\left(x_{L}^{n}\right)\right|\right|}_{2}^{2}+k\cdot {\left|\left|x_{L}^{n}-{\bar{x}}_{L}\right|\right|}_{2}^{2}\right)$$

where $m\left(x_{L}^{n}\right)$ is the prediction of sample $x_{L}^{n}$, ${\bar{x}}_{L}$ is the mean of sample $x_{L}^{n}\;\mathrm{and}\;k$ is a hyperparameter. Here, ${\left|\left|x_{H}^{n}-m\left(x_{L}^{n}\right)\right|\right|}_{2}^{2}$ is the label space operation and $k\cdot {\left|\left|x_{L}^{n}-{\bar{x}}_{L}\right|\right|}_{2}^{2}$ is the data space operation (different k values produce different results as discussed in the next section). This regularization (similar to the $E\left(X_{L},X_{H}\right)$ in Equation (15)) can simplify the calculation of the linear regression model $m\left(x_{L}^{n}\right)$. After the data in the current node are split and forwarded to the left and right child nodes, respectively, the tree continues to grow until the last leaf node has been created. Finally, classification is accomplished through voting to determine the optimal solution.

### 3.3. The Method Scheme

**Figure 1:**
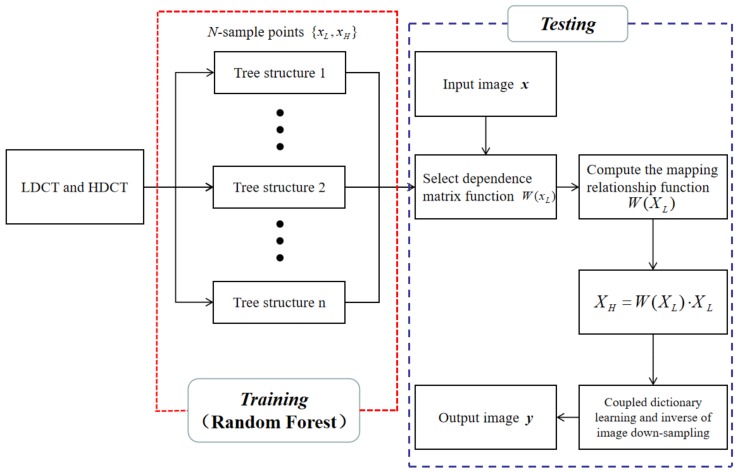
Flowchart of the SR algorithm.

This section provides a brief description of the logic in the proposed algorithm, both the basic scheme for SR and the tree-structure construction algorithm for the random forest. These basic schemes are summarized in Table 1 and Figure 1 for clarity.

The first stage is the training stage (the red block). In this module, using the LDCT image and the corresponding HDCT image as a training set, according to the third section, a decision tree is generated by the training set and a random forest is trained to find the mapping relationship $W\left(X_{L}\right)$ between the two images. The second stage is the test stage (the blue block). A non-training set LDCT image is used as the input image and using the developed mapping function and the LDCT image matrix $X_{L}$, the new image matrix $X_{H}$ is reconstructed. Finally, the coupled dictionary learning of $D_{L}$ and $D_{H}$ is performed according to Equation (5), the inverse process of image down-sampling is performed according to Equation (4) to obtain the final reconstructed image.

Steps 3 and 4 of Table 1 mention training an individual tree and a random forest. Table 2 provides the algorithm for generating the random forest.

## 4. Experiments and Results

In this section, experiments based on clinical data are performed using the proposed random forest solution for SR. All the experiments were executed in MATLAB 2016a on an Ubuntu 18.04 operating system with an Intel^®^ Core^TM^ i5-7500 CPU @ 3.40 GHz and 64.0 GB of RAM.

All the CT images in the following experimental sections were provided by the ***United Imaging*** company. For this experiment, 100 LDCT images and the corresponding HDCT images are selected as low-resolution image training sets and high-resolution image training sets, respectively, for training and the mapping relationship is determined. This step constitutes the training phase. Here, HDCT denotes a full-dose CT image and LDCT denotes a quarter-dose CT image. In the testing phase, a non-training set LDCT image is used as the input image, combined with the training mapping relationship and then, a new CT image is obtained by reconstructed by coupled dictionary learning. Finally, the CT image reconstructed by the method of the present invention is compared with the input LDCT image, the original HDCT image and the image reconstructed by the conventional interpolation method. The findings prove that the proposed method has strong robustness in reducing noise and artifacts.

### 4.1. Experimental Parameters and Evaluation Function

In the experiment, the main parameters include the number of trees T in the system, the maximum tree depth $\xi_{max}$, the regularization parameter λ for linear regression in the leaf nodes and the regularization parameter k of the last splitting target. When no special values are provided, the above parameters are set to T=10, $\xi_{max}=15$, η=0.01 and k=1.

The resulting reconstructed image was evaluated using the ***peak signal-to-noise ratio (PSNR)*** and the ***structural similarity index measurement (SSIM)*** of the image as evaluation criteria.

The definition of *PSNR* is as follows:

(16) $$PSNR=10\times \mathrm{lg}\left(\frac{255^{2}}{MSE}\right),\;MSE=\frac{\left(\sum_{j=1}^{height}\sum_{i=1}^{width}{\left(I_{orig}\left(i,j\right)-I_{tar}\left(i,j\right)\right)}^{2}\right)}{height\times width}$$

where MSE is the mean square error, height and width are the height and width of the image, respectively, $I_{orig}$ is the source image and $I_{tar}$ is the image to be evaluated. The PSNR reflects the loss of high-frequency components from the image: higher PSNR values indicate smaller loss and a better reconstruction effect.

The *SSIM* is defined as follows [32]:

(17) $$SSIM\left(x,y\right)=\frac{\left(2u_{x}u_{y}+C_{1}\right)\left(2\sigma_{xy}+C_{2}\right)}{\left(u_{x}^{2}+u_{y}^{2}+C_{1}\right)\left(\sigma_{x}^{2}+\sigma_{y}^{2}+C_{2}\right)}$$

where $u_{x}$, $u_{y}$ and $\sigma_{x}$, $\sigma_{y}$ are the mean and standard deviation of the image at *x*, *y*, respectively; $\sigma_{xy}$ is the covariation of *x*, *y*; and $C_{1}$ and $C_{2}$ are constants (set to 1 in the experiment). The SSIM is the structural similarity index of *x* and *y* images and is used to measure the similarity between two images. The SSIM is more similar to the human eye’s evaluation of image quality; its value ranges from 0 to 1. The closer the SSIM value is to 1, the more similar the two images are.

### 4.2. Clinical Data Experiments

**Figure 2:**
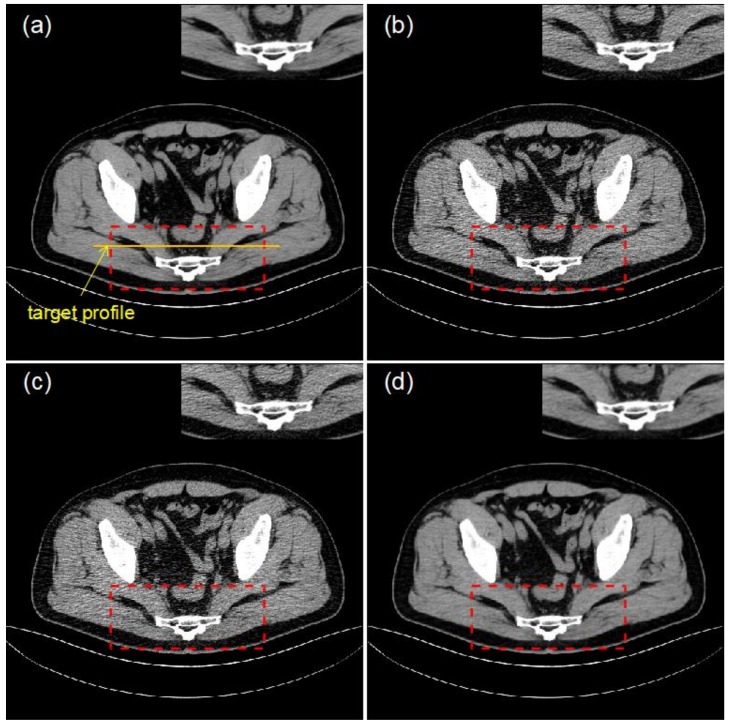
From left to right, top to bottom: (**a**) HDCT image; (**b**) LDCT image; (**c**) reconstructed image obtained using the bicubic interpolation method; (**d**) image reconstructed by the proposed method.

In this experiment, clinical data were used for validation and to test the performance and robustness of the proposed method. Taking a low-dose CT image of the non-training set as input, the method proposed in this paper and the bicubic interpolation method are applied to reconstruct the input CT images. Figure 2 compares the image quality between the two methods based on the *PSNR* and *SSIM* metrics mentioned in the previous section. Figure 2a shows the original image, a high-dose CT (HDCT) image, for reference and Figure 2b shows the corresponding input image, a low-dose CT (LDCT) image. Figure 2c shows the image reconstructed by the bicubic interpolation method (***PSNR*** = 25.37 dB, ***SSIM*** = 0.79) and Figure 2d shows the image reconstructed by the method proposed in this paper (***PSNR*** = 35.94 dB, ***SSIM*** = 0.91). The improvements in the two image quality indexes when using the proposed method are clear: a 41.66% improvement in PSNR and a 15.19% improvement in SSIM. These results demonstrate that the proposed method achieves significant improvements in image high frequency retention, denoising and image reconstruction quality compared with a traditional interpolation method.

**Figure 3:**
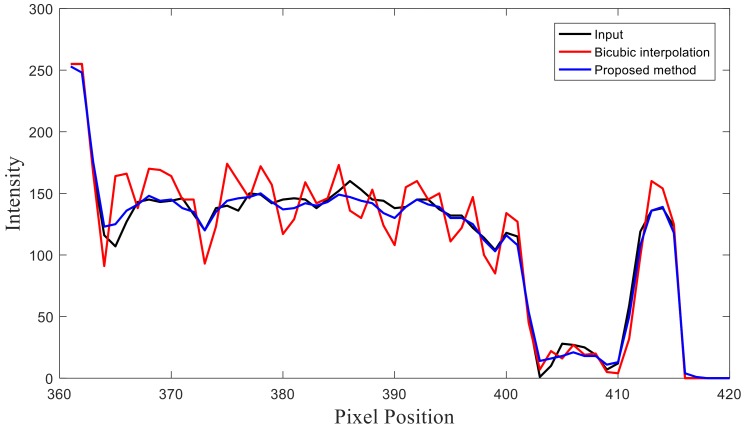
Profiles of different results are shown for the 320th row of the image in Figure 2. The black curve represents the profile of the original CT image in Figure 2a. The red curve represents the profile of the reconstructed CT image obtained using the bicubic interpolation method in Figure 2c. The blue curve represents the profile of the reconstructed CT image obtained using the proposed method in Figure 2d.

**Figure 4:**
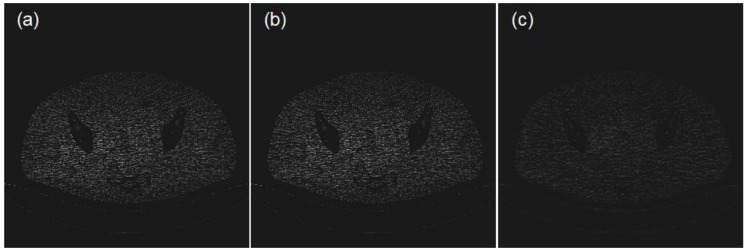
From left to right, (**a**–**c**) respectively represent the residual image of the LDCT image in Figure 2b, the reconstructed results by the bicubic interpolation in Figure 2c and the method proposed in this paper in Figure 2d.

The profile and residual images are also compared in Figure 3 and Figure 4. It can be concluded that the effect and performance of the proposed method in image reconstruction is superior to those of the traditional bicubic interpolation method.

**Figure 5:**
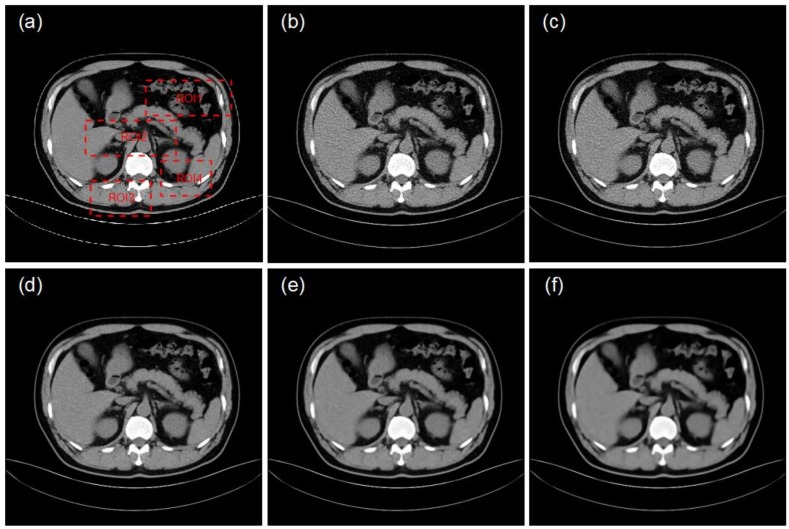
(**a**) HDCT image; (**b**) LDCT image; (**c**) Reconstructed image obtained using the bicubic interpolation method; (**d**) The image reconstructed by the method of this paper with 1 iteration; (**e**) The image reconstructed by the method of this paper with 2 iterations; (**f**) The image reconstructed by the method of this paper with 5 iterations.

**Figure 6:**
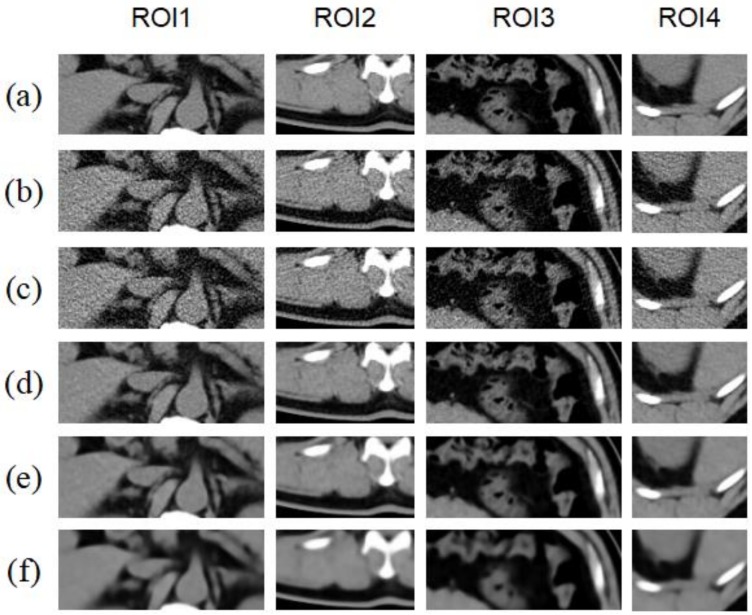
Images (**a**–**f**) show zoomed images of the portions marked with red squares in Figure 5a, providing more detail of the differences in reconstructed image quality under different iterations.

Different numbers of iterations were employed in the proposed method and the reconstructed images obtained in Figure 3 were compared. To be more convincing, three representative parts were selected for comparison in Figure 5 and Figure 6 and the related data are shown in Table 3, Table 4 and Table 5.

**Figure 7:**
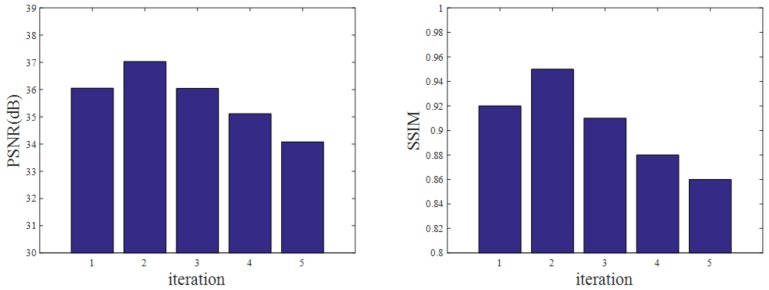
Changes in PSNR and SSIM values with the number of iterations for the simulation experiment using the proposed method.

As shown in Figure 7a,b and Table 3, Table 4 and Table 5, the image quality clearly changes as the number of iterations changes. The correlation image quality parameters PSNR and SSIM are optimal when iterating twice, after which these parameters have a downward trend.

### 4.3. Parameter Evaluation

According to the analysis in Section 2, factors that affect the random forest include the objective function for evaluating the potential segmentation function and the inherent randomness. Therefore, during the statistical analysis of the reconstruction results, two factors are considered here: the number of trees T in the random forest and the maximum depth of each tree structure $\xi_{max}$.

To control the variables and ensure authenticity during this experiment, all the following experiments involve only one iteration.

Random forest classifiers function similarly to voting. The construction of random forest classifiers [27] involves first generating a decision tree; then, multiple decision trees form the random forest. Each decision tree functions as a ballot; all the trees vote to yield the final result. A larger number of trees tends to produce a better final result but increases the time required to reach a final decision; therefore, an optimal solution must be found. Here, $\xi_{max}=15$ is set as the default.

**Figure 8:**
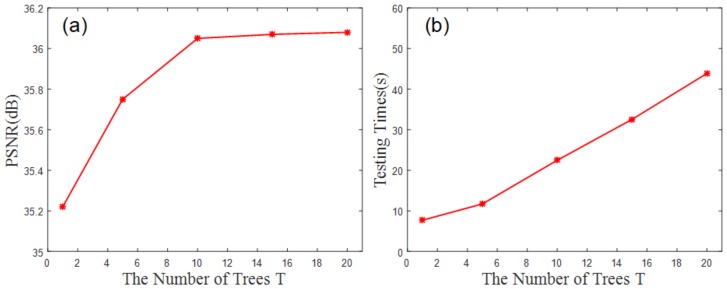
As shown in (**a**), when T=10, the PSNR is close to saturated; in (**b**) the time increases linearly as T increases.

Figure 8a shows the effect of the parameter T on the experiment. The *PSNR* value increases steadily and eventually becomes saturated as T increases. As shown in Figure 8a, the PSNR is saturated when T=10. Figure 8b shows the relationship between the number of trees T and the total calculation time.

According to the graphs in Figure 8, it can be concluded that T=10 is optimal, that is, the algorithm achieves good results and completes in a reasonable amount of time when 10 trees are used.

After determining the optimal number of trees (T=10), the maximum depth of each decision tree can be discussed. Decision tree classification starts from the root node, classifies new subnodes according to their characteristics and then classifies the subnodes as new root nodes; consequently, the subclasses are sorted down to the maximum depth to obtain the final result. The maximum depth principle is the same as that for the number of trees: greater depth provides a better classification effect but requires more time to generate the tree. Therefore, finding the best solution for tree depth is also crucial.

**Figure 9:**
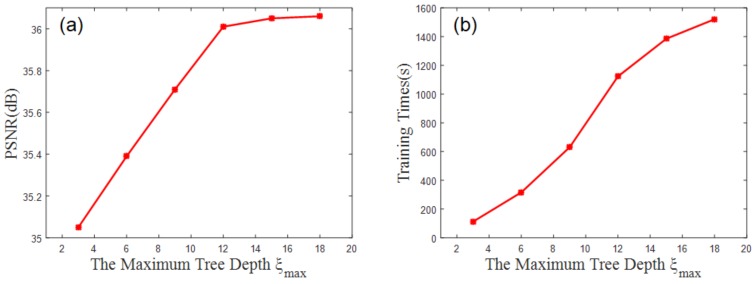
(**a**) shows that when $\xi_{max}=15$, the result is saturated; (**b**) shows the relationship between the maximum depth of the tree $\xi_{max}$ and the training time.

Figure 9a shows the relationship between the maximum tree depth $\xi_{max}$ and the experimental outcome. Tree depth has a strong influence on the training. Figure 9a shows that a steady state is reached when the depth $\xi_{max}=15$, that is, the selected sample image is saturated. This relationship is reflected by Equation (15), which directly affects the training of LDCT and HDCT images. Figure 9b shows the relationship between the maximum depth of the tree $\xi_{max}$ and the training time. It is concluded that the maximum depth of the tree is $\xi_{max}=15$.

**Figure 10:**
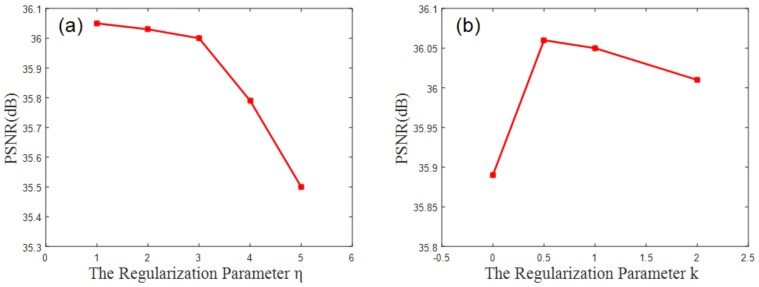
(**a**) The effect of the regularization parameter η on the results; (**b**) the effect of the regularization parameter k on the results.

The regularization parameter η of the linear regression in the leaf node mentioned in Section 2.2 and the regularization parameter k of the splitting target mentioned in Equation (15) also have a certain influence on the final random forest result but their influences are not as obvious as those of the first two factors; consequently, comparisons are provided herein but detailed explanations are omitted. As shown in Figure 10a, when $\eta >10^{-2}$, the declining PSNR trend is obvious and in Figure 10b, a k value between 0.5 and 1 is most appropriate; that is, the PSNR value remains the highest within this interval.

## 5. Conclusions

In this paper, a new method for low-dose CT image SR reconstruction is proposed that avoids using sparse coding dictionaries to learn the mapping from LR images to HR images, as in general sparse representation of compressed sensing. Instead, the problem of mapping HDCT image blocks to LDCT image blocks is solved by using a random forest and combined with coupled dictionary learning to complete LDCT image reconstruction. CT images acquired from various parts of the human body have similar features and therefore, CT images of different parts of the body are included in the training set. To obtain a better reconstruction effect for a specific part of the test, CT images of that specific body part can be used as the training set. An iterative capability is also incorporated in this paper to improve the robustness of the method. Compared with traditional interpolation methods, the proposed method greatly reduces noise and artifacts. The algorithm proposed in this paper improves the resolution of noisy images and produces larger PSNR values and SSIM values. The method proposed in this paper can be applied in different CT fields, such as dual-source CT (DSCT) and can also be applied to other medical imaging fields, such as positron emission computed tomography (PET). In the training process, computer multithread computing is used to reduce the training time. Compared with the deep learning-based CT super-resolution reconstruction method, which is of great interest in the academic world, this method has a substantial advantage in terms of running time but cannot handle large training sets because of CPU and computer memory limitations. In the future, the method proposed herein will be combined with deep learning in the field of super-resolution imaging and a larger database will be trained to improve the reconstruction effect.

## Figures and Tables

**Table 1:** Basic Scheme for SR.

1 **Input:** an LDCT image ***x***
2 **Output:** the final processed image ***y***
3 LDCT image and HDCT image **N-sample points** $\left\{x_{L}^{n},x_{H}^{n}\right\}$ in the training set
4 **Train individual random forest trees** and then combine the trained trees into a random forest
5 **The dependence matrix function** $W\left(x_{L}\right)$ is obtained by Equation (10)
6 **Compute the mapping relationship function** $W\left(X_{L}\right)$ using Equations (7) and (8)
7 **The relationship** between the data matrix$X_{L}$$\;\mathrm{of}\;\mathrm{the}\;\mathrm{LR}\;\mathrm{and}\;\mathrm{the}\;\mathrm{data}\;\mathrm{matrix}\;X_{H}$ of the HR is obtained by Equation (6)
8 **Coupling dictionary learning** of the LR dictionary$D_{L}$$\;\mathrm{and}\;\mathrm{the}\;\mathrm{HR}\;\mathrm{dictionary}\;D_{H}$ is completed by Equation (5)
9 Implement the **inverse of image down-sampling** by Equation (4) and obtain the final image y by Equation (3)

**Table 2:** Tree construction for a random forest.

1 for ***k* = 1** to ***K***
2 **Randomly extract** N-samples to construct feature vector sets
3 while (tree depth is below the minimum)
(1) **randomly select *n*** eigenvectors from the set of feature vectors
(2) select the **optimal vector** and the **optimal split point** from the feature vectors
(3) split the optimal split **point into** left and right child **nodes**
(4) update tree depth
4 end while
$5\;\mathrm{create}\;a\;\mathrm{tree}\;T_{k}\left(x\right)$
6 end for
$7\;\mathrm{return}\;\mathrm{the}\;\mathrm{collection}\;\mathrm{of}\;\mathrm{trees}\;{\left\{T_{k}\left(x\right)\right\}}_{k=1}^{K}$

**Table 3:** Comparison of relevant data.

	LDCT	Bicubic	RFSR	RFSR 2nd	RFSR 5th
***PSNR*** **(dB)**	21.65	26.23	36.05	37.03	34.08
***SSIM***	0.75	0.80	0.92	0.95	0.86

**Table 4:** The ***PSNR*** value of the four ROIs marked by red squares in Figure 5a.

*ROI*	LDCT	Bicubic	RFSR	RFSR 2nd	RFSR 5th
**1**	20.55	25.76	35.89	36.97	34.01
**2**	21.33	26.13	35.97	37.01	34.03
**3**	22.31	27.43	36.12	37.09	34.09
**4**	22.06	26.54	36.45	37.63	34.61

**Table 5:** The ***SSIM*** value of the four ROIs marked by red squares in Figure 5a.

*ROI*	LDCT	Bicubic	RFSR	RFSR 2nd	RFSR 5th
**1**	0.71	0.79	0.90	0.92	0.86
**2**	0.74	0.81	0.92	0.95	0.88
**3**	0.78	0.83	0.91	0.94	0.87
**4**	0.76	0.82	0.89	0.93	0.85
